# Supplementary material for: An improved genome assembly uncovers prolific tandem repeats in Atlantic cod
Source: BMC Genomics. 2017 Jan 18;18:95. doi: 10.1186/s12864-016-3448-x (PMC5241972; doi:10.1186/s12864-016-3448-x)
Supplement: Additional file 1 — Supplementary figures and tables. (PDF 647 kb) [file 12864_2016_3448_MOESM1_ESM.pdf]

Additional file

An improved genome assembly uncovers prolific tandem repeats in Atlantic cod

Ole K. Tørresen[1†], Bastiaan Star[1], Sissel Jentoft[1,2], William Brynildsen Reinar[1], Harald Grove[3], Jason R. Miller[4], Brian P. Walenz[5], James Knight[6], Jenny M. Ekholm[7], Paul Peluso[7], Rolf B. Edvardsen[8], Ave Tooming-Klundrerud[1], Morten Skage[1], Sigbjørn Lien[3], Kjetill S. Jakobsen[1] and Alexander J. Nederbragt[1,9†]

Addresses:
[1]Centre for Ecological and Evolutionary Synthesis (CEES), Department of Biosciences, University of Oslo, NO-0316 Oslo, Norway.
[2]Department of Natural Sciences, University of Agder, NO-4604 Kristiansand, Norway.
[3] Centre for Integrative Genetics (CIGENE), Department of Animal and Aquacultural Sciences, Norwegian University of Life Sciences, NO-1432, Ås, Norway.
[4] J. Craig Venter Institute, 9704 Medical Center Drive, 20850, Rockville, MD, USA.
[5] Genome Informatics Section, Computational and Statistical Genomics Branch, National Human Genome Research Institute, National Institutes of Health, 20892, Bethesda, MD, USA.
[6] Yale School of Medicine, Yale University, 06520, New Haven, CT, USA.
[7] Pacific Biosciences, Menlo Park, CA, USA.
[8] Institute of Marine Research, Nordnes, NO-5817, Bergen, Norway.
[9] Biomedical Informatics Research Group, Department of Informatics, University of Oslo, NO-0316 Oslo, Norway
[†]Corresponding authors: E-mail: o.k.torresen@ibv.uio.no, lex.nederbragt@ibv.uio.no

1 Supplementary Figures

Additional file 1: Figure S1: The frequency of tandem repeats in genome assembly, promoters and coding regions.
As Figure 5 in the manuscript, but plotting the number of TRs detected per Mbp assembly instead (frequency).

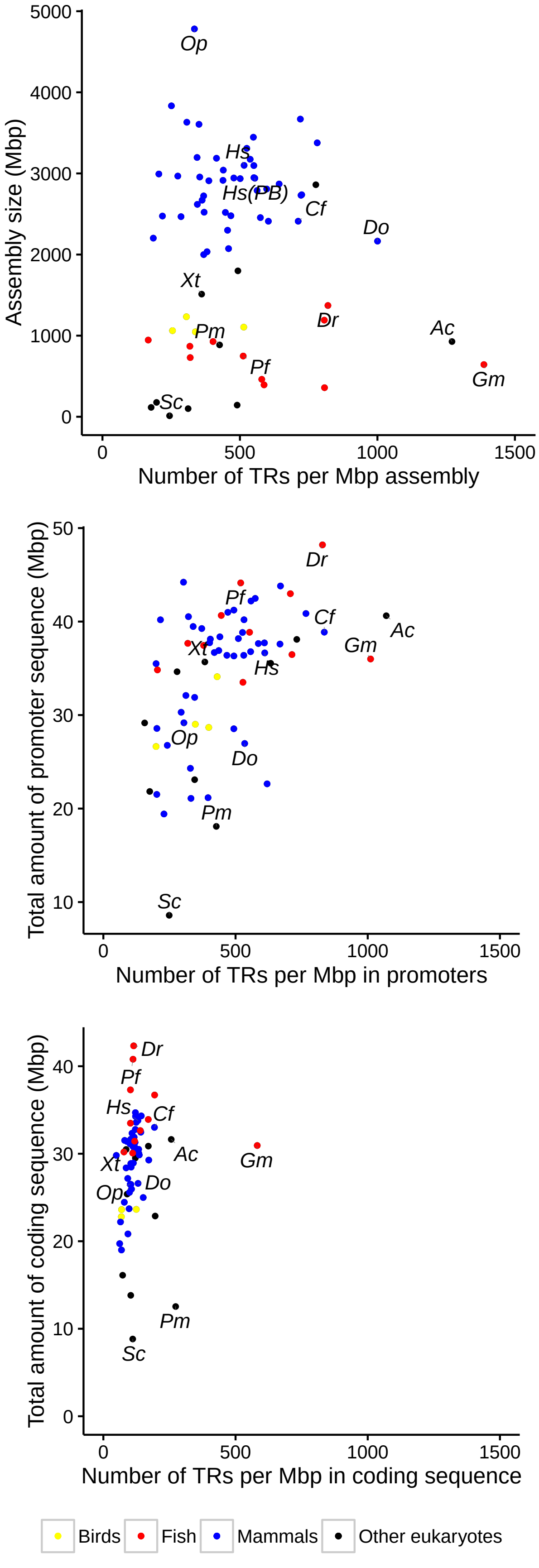

Additional file 1: Figure S2: The intersections between contig termini and different annotated features.
As Figure 6 in the manuscript, but these are absolute numbers and not percentages of the total.

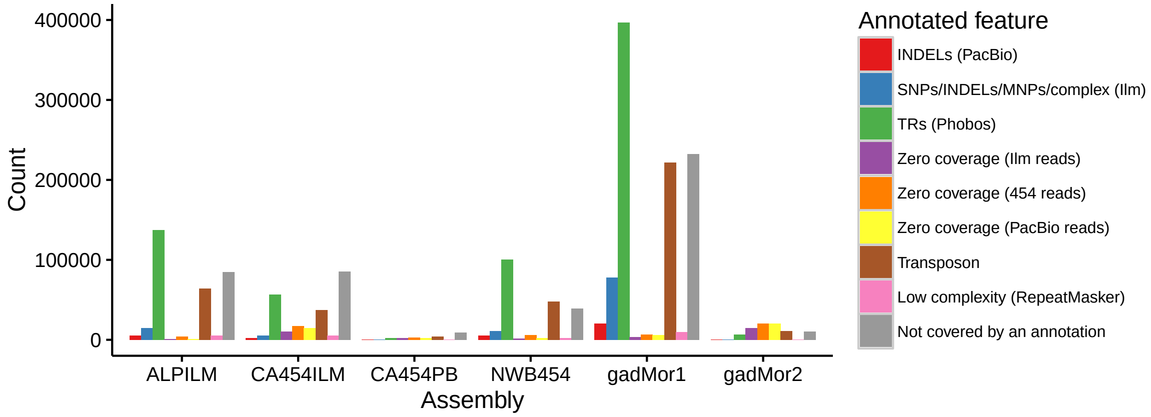

Additional file 1: Figure S3: The distribution of lengths of STRs in cod as found by lobSTR and Phobos.
Only repeats of unit size 1-6 bp and of total length longer than 13 bp are included.

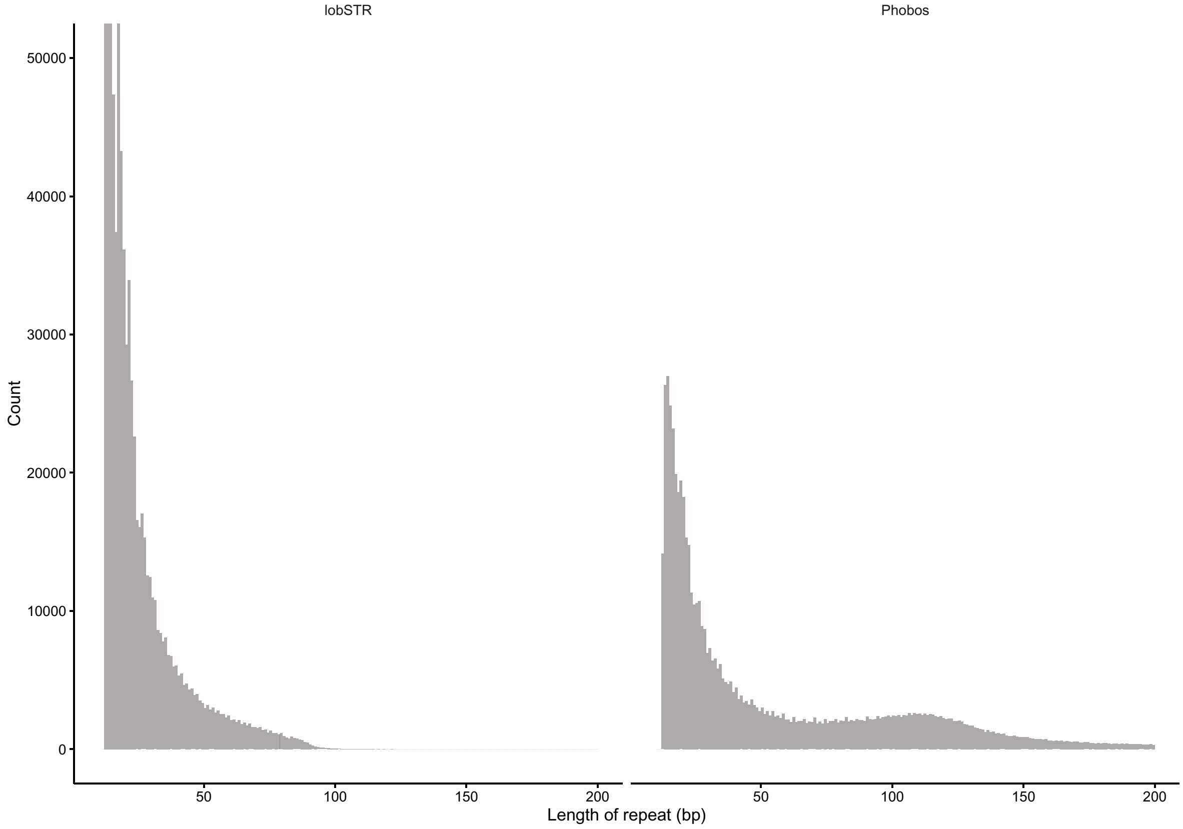

Additional file 1: Figure S4: Tandem repeats in genes.
Percentage of genes (vertical axis) with a certain number of tandem repeats (i.e.; 0, 1, 2, 4 or more individual repeats within the genes, horizontal axis) in selected species.

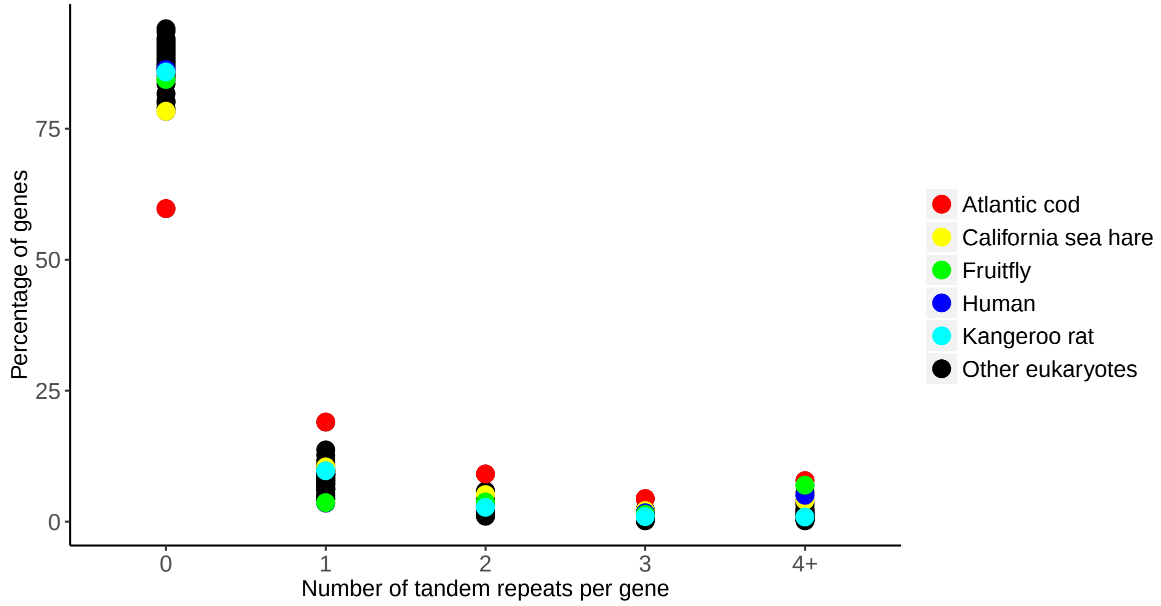

2 Supplementary Tables

Additional file 1: Table S1: Read datasets, accession numbers and amount.

Technology	Insert size (bp)	Average read length (bp)	Amount bases (Gbp)	Number of pairs	Total coverage	APLILM	NEWB454	CA454ILM	CA454PB
Illumina	180	100	97	485,469,807	105x	52x		25x	25x
Illumina	300	100	116	580,675,602	140x	140x		6x	
Illumina	5000	100	103	513,197,070	124x	124x		124x	
454	1000	170	0.4	1,185,540	0.6x		0.6x	0.6x	0.6x
454	1400	170	0.3	939,375	0.5x		0.5x	0.5x	0.5x
454	1800	175	0.4	1,174,856	0.6x		0.6x	0.6x	0.6x
454	2300	160	0.5	1,424,229	0.7x		0.7x	0.7x	0.7x
454	3000	170	1.2	3,627,219	1.9x		1.9x	1.9x	1.9x
454	8000	175	1.4	3,876,715	2.1x		2.1x	2.1x	2.1x
454	20000	200	0.3	818,578	0.5x		0.5x	0.5x	0.5x
454	Not paired	340	23.6	NA	36.3x		36.3x	36.3x	
PacBio (C2C2)	Not paired	2400	7.1	NA	11.0x				11.0x
PacBio (C2XL)	Not paired	3500	2.3	NA	3.5x				3.5x
PacBio (XLXL)	Not paired	3800	2.8	NA	4.4x				4.4x
Sanger	100000	900	0.07	39,017	0.1x			0.1x	

Additional file 1: Table S2: Overview of assembly statistics. CEGMA annotates 458 highly conserved eukaryotic genes, REAPR analyses the discordance between the expected order, orientation and distance of mapped paired reads, with FRC^{bam} using a similar approach. Assemblies chosen for reconciliation in bold.

Assembly	Total size assembly (Mbp)	N50 contig (kbp)	N50 scaffold (Mbp)	Percentage gap bases	CEGMA	REAPR[1]	FRC^{bam}[2]	Potential conflict (sequences)[3]
ALPILM	660	4.4	0.16	28.7	424 (92.6 %)	19,787	2,182,096	122
+ Pilon	660	4.5	0.16	28.5	427 (93.2 %)	18,668	2,171,880	123
+ PBJelly	620	8.3	0.16	9.7	431 (94.1 %)	23,994	1,878,873	134
**+ PBJelly + Pilon**	**620**	**8.5**	**0.16**	**9.6**	**431 (94.1 %)**	**24,066**	**1,828,800**	**134**
NEWB454	656	6.2	1.30	24.4	435 (95.0 %)	18,117	2,044,008	26
+ Pilon	656	6.6	1.30	24.0	430 (93.9 %)	15,917	2,018,862	19
+ PBJelly	646	10.2	1.30	15.4	437 (95.4 %)	16,930	1,875,518	28
**+ PBJelly + Pilon**	**645**	**10.4**	**1.30**	**15.1**	**437 (95.4 %)**	**17,534**	**1,822,739**	**28**
CA454ILM	647	9.9	0.50	3.5	447 (97.5 %)	7,406	1,351,500	96
**+ Pilon**	**648**	**10.2**	**0.50**	**3.4**	**444 (97.0 %)**	**7,025**	**1,339,572**	**83**
+ PBJelly	672	15.3	0.52	2.5	447 (97.5 %)	14,755	1,449,619	98
+ PBJelly + Pilon	673	15.6	0.52	2.5	444 (97.0 %)	14,750	1,438,035	92
CA454PB	682	95	0.27	1.62	431 (97.6 %)	8,617	1,508,054	188
+ Pilon	683	95	0.27	1.6	441 (96.3 %)	7,754	1,426,588	163
+ PBJelly	687	96	0.27	1.1	436 (95.2 %)	8,565	1,502,582	163
**+ PBJelly + Pilon**	**684**	**97**	0. **27**	**1.1**	**439 (95.6 %)**	**9,043**	**1,418,020**	**165**

[1] detected potential errors, fewer is better
[2] total number of features (i.e., potential assembly problems), fewer is better
[3] number of sequences mapping to more than one linkage group or to multiple linkage groups, fewer is better

Additional file 1: Table S3: Linkage groups and their sizes.

Linkage group	Size (bp)
1	28,303,952
2	24,054,406
3	29,451,055
4	34,805,322
5	24,074,055
6	25,464,620
7	31,232,877
8	26,796,886
9	25,382,314
10	25,304,306
11	28,942,968
12	27,297,974
13	25,676,735
14	29,296,932
15	26,597,959
16	31,093,243
17	19,149,207
18	22,554,255
19	21,176,260
20	24,149,133
21	22,510,304
22	21,735,703
23	23,264,654
Unplaced	46,128,564

Additional file 1: Table S4: Calculating of genome size using odd-sized kmers from 17 through 31 with SGA PreQC.

k	Estimated genome size (bp)
17	633,173,903
19	617,492,869
21	615,747,892
23	621,292,036
25	612,150,017
27	606,607,539
29	601,318,671
31	597,207,477
